# Utilizing the Human Animal Bond to Promote Preventive Care Engagement in Underserved Communities: A Descriptive Study of 2 U.S. One Health Clinics

**DOI:** 10.1177/21501319251369270

**Published:** 2025-09-09

**Authors:** Kimberly Aguirre Siliezar, Sonny Patel, Reema Chande, Alaina Joiner, MacKenzie C. Hoover, Mary W. Mathis, Janet Hendrickson, Julio Siliezar, Kristin Jankowski

**Affiliations:** 1 University of California Davis School of Veterinary Medicine, Davis, CA, USA; 2 Mercer University School of Medicine, Macon, GA, USA; 3 College of Liberal Arts and Sciences, Mercer University, Macon, GA, USA; 4 People & Pets Project, Macon, GA, USA; 5 Department of Internal Medicine, University of California Irvine School of Medicine, Irvine, CA, USA; 6 One Health Institute, University of California Davis School of Veterinary Medicine, Davis, CA, USA

**Keywords:** One Health Clinic, human-animal bond, underserved communities, access to care, healthcare model

## Abstract

**Introduction/Objectives:**

The purpose of this descriptive study was to strengthen understanding of the human-animal bond and the impact of One Health Clinics (OHCs) on the communities they serve. We aimed to assess how joint access to veterinary care and human health services enables community members to engage with healthcare for themselves.

**Methods:**

Individuals attending 2 OHCs in the United States were surveyed to gain insight into reasons for attending OHCs, attitudes on the human-animal bond, healthcare access and utilization, and pet owner satisfaction and trust toward medical and veterinary professionals. Both clinics operated in areas with limited medical and veterinary healthcare access, but varied in clinic structure and availability of human healthcare volunteers.

**Results:**

A total of 175 surveys were analyzed. Most participants attended primarily for veterinary services (Clinic A: 91%, Clinic B: 75%). However, a significantly higher proportion of Clinic B participants received health screenings (91% vs 32%, *P* < .0001), learned new health information (48% vs 31%, *P* = .0129), and were provided with follow-up health resources (84% vs 51%, *P* = .000007). Across both sites, the majority expressed high trust in medical and veterinary providers (Clinic A: 84%-95%, Clinic B: 90%-98%) and reported high satisfaction with the care received. Most participants considered their pets vital family members (Clinic A: 81%, Clinic B: 93%), and many credited their pets with supporting physical activity and reducing feelings of depression and loneliness. Importantly, a majority of respondents at both clinics indicated they were more likely to seek healthcare for themselves if veterinary services were also available (Clinic A: 56%, Clinic B: 72%).

**Conclusions:**

These findings suggest that OHCs have the potential to enhance access to human healthcare in underserved communities by utilizing veterinary services as a point of engagement. Participants reported a strong bond with their pet. Based on reported levels of trust and satisfaction, the OHC model may help strengthen relationships between underserved communities and human healthcare and veterinary professionals.

## Introduction

Despite ongoing efforts to improve access to medical and veterinary care, significant barriers limit people’s ability to receive healthcare for themselves and their pets.^1,2^ Individuals may prioritize their pet’s needs and health concerns above their own.^1^ Many pet owners will do more for their pets than for themselves, including holding off on seeking healthcare for themselves if they lacked social support or alternative care options.^3,4^ Because the well-being of people and their pets is closely connected, reducing barriers to care and embracing the power of this connection may improve healthcare access by encouraging individuals to seek care for both their pet and themselves.^5^

“Bonded families” refers to the deep connection between human and animal family members, highlighting the emotional attachment humans have with their pets.^6,7^ Many families face challenges in accessing veterinary care due to costs, provider availability, and veterinary staff shortages.^8-10^ Lower-income families are particularly affected, often making pet care decisions based on cost combined with limited access.^11^ National efforts have been made to address these barriers in veterinary and human healthcare settings.^6^ The human-animal bond (HAB), a mutually beneficial relationship between humans and non-human animals, is influenced by an individual’s mental, physical, and social well-being.^12^ The HAB has been shown to enhance the health of both parties by reducing stress and improving cardiovascular health.^12-14^ Additionally, pets provide psychological stimulation that encourages humans to care for themselves, making them 4 times more likely to meet physical activity guidelines.^13,15^ However, if pets are unwell and lack veterinary care, it can cause significant emotional distress for their human counterparts, who may avoid seeking medical care for themselves if pet care is unavailable.^5,6,16,17^

The One Health High-Level Expert Panel (OHHLEP) defines One Health as “an integrated, unifying approach that aims to sustainably balance and optimize the health of people, animals, and ecosystems.”^18^ The One Health Clinic (OHC) approach identifies the commonalities between humans and animals and simultaneously aims to meet the unique health needs of both groups in a single setting. This healthcare model encourages collaboration between various healthcare professionals and community members to create an environment that reduces barriers and integrates the core tenets of human, environmental, and animal health. Compared to traditional methods for delivering health resources, this model is defined by a sustained, voluntary relationship with the community, building patient-provider trust, interprofessional proximity, and reducing barriers to accessing healthcare.^19,20^

Our aim is to strengthen our understanding of the HAB and OHCs’ community impact and to assess how joint access to veterinary services and human healthcare enables community members to engage with healthcare for themselves. Through our analysis, we hope to (1) confirm that OHCs provide services to underserved communities, (2) determine if OHCs are associated with high levels of trust and satisfaction toward medical and veterinary healthcare professionals, (3) assess the HAB for individuals attending OHCs, (4) assess attendee preference of this model of healthcare delivery, and (5) assess the utilization of healthcare services by individuals at these clinics.

## Methods

Community members attended 2 OHCs in the United States between August 2023 and November 2024.

Clinic A, located in a western state, serves a rural, agricultural community with a population of approximately 1000, where 18.9% of the population lives in poverty and the majority identifies as Hispanic/Latino.^21^ There is a notable migrant worker population within this area, most of whom are undocumented immigrants. There is limited access to human medical care in this area, and to date, a veterinary clinic has never been established in the community.^19^ The nearest medical and veterinary clinics are located 10 miles away. The clinic takes place once a month and primarily offers preventative care and general wellness services for small domestic animals, including vaccinations, parasite preventatives, chronic disease medications, and minor surgical procedures. Additionally, every 2 out of 3 monthly clinics, nursing students and preceptors offer pet owners health screenings such as blood glucose and blood pressure checks, and provide resources on managing chronic conditions and accessing local care. Surveys were conducted only when medical volunteers were present at Clinic A.

Clinic B is located in a southeastern state, in a mid-sized city with a population of approximately 156 000. Approximately 54.3% identify as Black or African American, and close to 25% of residents live in poverty.^22^ The need for consistent primary care is evident as rates of physical distress, diabetes, HIV, obesity, mental distress, and loneliness are all higher than at the state and national levels.^23^ While health care and veterinary services are available in the region, residents have limited access due to provider availability and care costs. This community faces significant socioeconomic challenges, including a child poverty rate of 32%, and lower health literacy and educational attainment compared to state and national averages in 2025.^23^ Clinic B offers monthly pop-up clinics providing free veterinary wellness services and vaccines to pets after their owners receive free basic health screenings. At each clinic, medical students and preceptors provide blood glucose and blood pressure checks for pet owners and offer guidance on accessing local health resources, such as low-cost clinics and medication counseling. The clinic aims to address the needs of underserved communities by combining pet care with support for owner health and access to community services.

While both clinics offer human health care services, attendees at Clinic B were required to receive, at a minimum, health counseling and resource information to receive veterinary care. Clinic A did not require engagement with human healthcare services to receive veterinary care, based on a belief that access to veterinary care should remain unconditional to maintain trust and encourage participation among community members who may be hesitant to engage with human health services. Clinic B did require engagement in a brief health assessment because of its foundational commitment to integrating preventive human healthcare into its service model.

All patients attending the clinic on designated days were invited to participate in the study; however, not all chose to complete the survey. Participation rates were not recorded. Those who agreed were administered a 32-question survey after providing either verbal or written consent, per each site’s institutional review board (IRB) requirements. The survey gathered information on demographics, the human-animal bond (HAB), healthcare access, and levels of satisfaction and trust in both medical and veterinary providers. It included multiple-choice questions, Likert-scale items, and 1 open-ended response. At Clinic A, surveys were administered on iPads using the Qualtrics^XM^ online platform. At Clinic B, surveys were completed on paper and later entered into Qualtrics^XM^. Only individuals aged 18 or older were eligible to participate, and no participant was surveyed more than once. No incentives or compensation were provided. The survey was available in both English and Spanish to accommodate the primary languages spoken by clinic attendees. No other language-related issues were reported or encountered during data collection. Survey administrators were trained undergraduate and professional students supervised by members of the research team who had completed CITI training. Analyses including descriptive statistics, Chi-square tests, and T-tests were performed using Microsoft Excel Version 16.92 (24120731).

## Results

**Figure 1.**
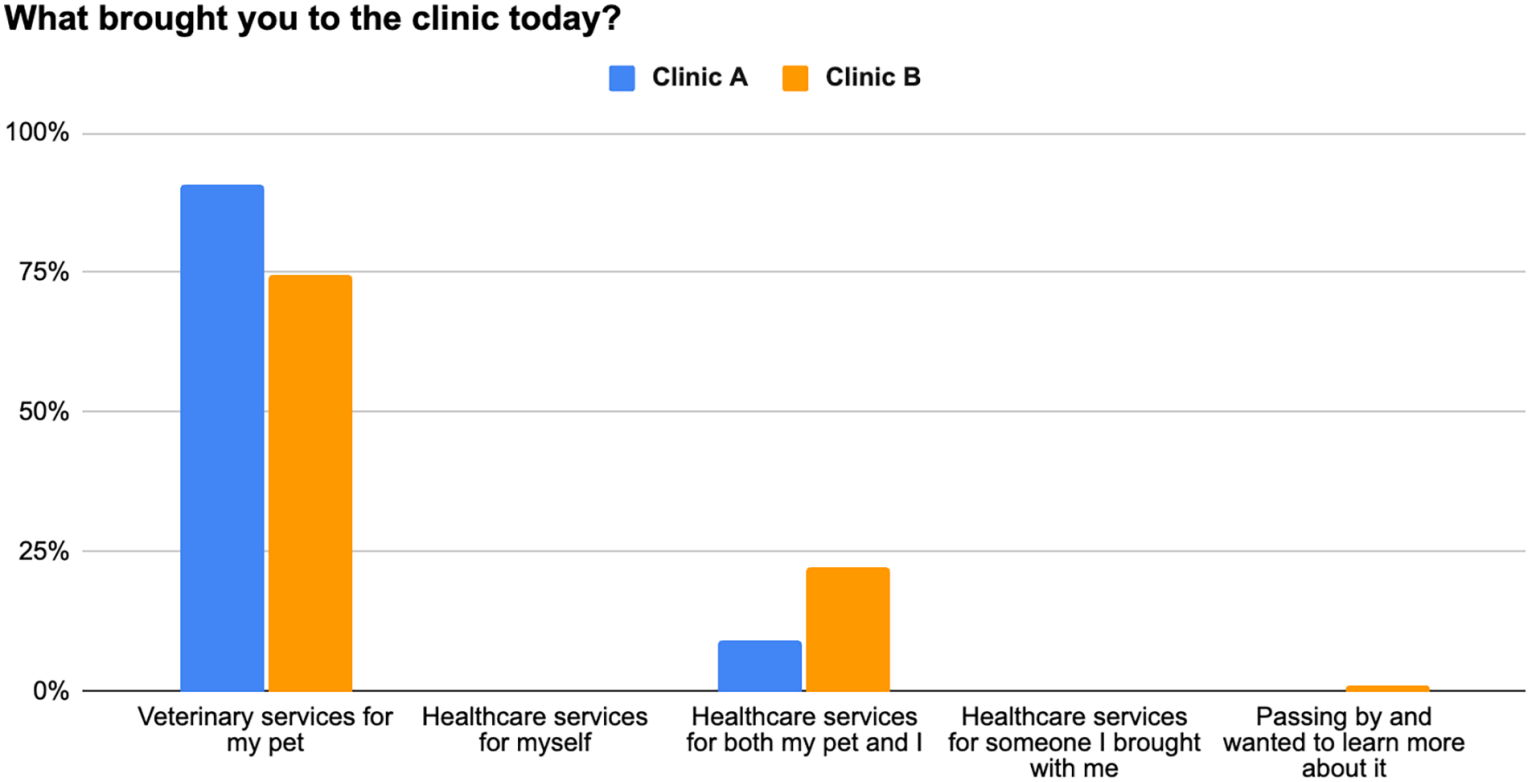
Reasons for attending 2 One Health clinics from 175 individuals between August 2023 and November 2024.

Between August 2023 and November 2024, 176 individuals completed the survey. One survey was removed during data analysis because the individual did not meet the age criterion, resulting in 175 surveys being analyzed for this study. Table 1 includes demographic information for the 175 individuals. At Clinic A, most participants identified as Hispanic/Latino (68%) and female (58%). At Clinic B, most participants identified as White (45%) or Black (41%) and female (84%). The majority of individuals at both Clinics A (76%) and B (81%) reported an annual family income level between $0 and $60 000. When asked for their primary reason for attending the clinic that day, 91% and 75% of participants from Clinic A and Clinic B, respectively, responded that they were there to receive veterinary services for their pets (Figure 1). In contrast, 9% and 22% (Clinic A and B, respectively) indicated they were there to receive healthcare services for both themselves and their pets.

**Table 1.** Demographic Information for 175 Individuals Seen at 2 One Health Clinics Between August 2023 and November 2024.

	Knights Landing, CA (N = 76) (%)	Macon, GA (N = 99) (%)	Total (N = 175) (%)
Age			
Mean	49	43	46
Range	21-78	19-81	19-81
Sex			
Female	44 (58)	83 (84)	127 (73)
Male	31 (41)	15 (15)	46 (26)
NA#	1 (1)	1 (1)	2 (1)
Race/ethnicity			
African American/Black	0	41 (41)	41 (23)
Asian	1 (1)	1 (1)	2 (1)
Latino/Hispanic	51 (67)	10 (10)	61 (35)
Native American	1 (1)	0	1 (1)
White	21 (28)	45 (45)	66 (38)
Other	2 (3)	2 (2)	4 (2)
Annual family income			
0-$20 000	19 (25)	30 (30)	49 (28)
$20 001-$40 000	26 (34)	26 (26)	52 (30)
$40 001-$60 000	12 (16)	25 (25)	37 (21)
$60 001-$80 000	10 (13)	9 (9)	19 (11)
$80 001-$100 000	4 (5)	5 (5)	9 (5)
$100 000+	2 (3)	2 (2)	4 (2)
NA#	3 (4)	2 (2)	5 (3)

NA#, left blank.

Table 2 presents responses from participants regarding human healthcare service utilization at both OHCs and satisfaction and trust toward veterinary and medical healthcare providers. At Clinic B, 91% of respondents reported having health screenings during their visit, and at Clinic A, 32%. At Clinics A and B, 31% and 48%, respectively, of individuals reported receiving information regarding their health that they previously were unaware of. Most participants (Clinic A = 57%, Clinic B = 75%) stated they would follow up with their healthcare provider regarding their health concerns. The majority of participants at both clinics responded that they were satisfied with the care they received from human healthcare providers (Clinic A = 80%, Clinic B = 98%) and veterinary providers (Clinic A = 96%, Clinic B = 93%), either “all the time” or “most of the time.” Participants also reported that they trusted human healthcare (Clinic A = 84%, Clinic B = 90%) and veterinary professionals (Clinic A = 95%, Clinic B = 98%) to make the best medical decisions for them, either “all of the time” or “most of the time.”

**Table 2.** Responses to Questions Regarding Use of Human Health Services, as Well as Satisfaction and Trust in Veterinary and Medical Care Providers, for 175 Individuals Seen at 2 One Health Clinics Between August 2023 and November 2024.

	Clinic A (N = 76) (%)	Clinic B (N = 99) (%)	Total (N = 175) (%)
Did you have any health screenings during your visit today?			
Yes	25 (33)	90 (91) *	115 (66)
No	49 (64)	9 (9)	58 (33)
NA#	2 (3)	0	2 (1)
Did you receive any information regarding your health that you may not have been aware of, such as having high blood pressure?			
Yes	24 (32)	48 (48) **	72 (41)
No	50 (66)	45 (45)	95 (54)
NA#	2 (3)	6 (6)	8 (5)
Were you given any follow-up information on resources for your health?			
Yes	36 (47)	78 (79) ***	114 (65)
No	38 (51)	18 (18)	56 (32)
NA#	2 (3)	3 (3)	5 (3)
Do you plan to follow up with a healthcare provider about your health needs/ concerns?			
Yes, I plan to follow up with my provider	44 (58)	74 (75)	118 (67)
I’d like to but I don’t know where to go	5 (7)	9 (9)	14 (8)
No, I didn’t receive any information that required follow-up	16 (21)	14 (14)	30 (17)
I’m not sure if I will follow up with my provider at this time	9 (12)	1 (1)	10 (6)
NA#	2 (3)	1 (1)	3 (2)
Are you satisfied with the health care you receive from human health professionals at this clinic?			
Yes, all of the time	54 (71)	93 (94)	147 (84)
Most of the time	7 (9)	4 (4)	11 (6)
Sometimes	1 (1)	2 (2)	3 (2)
No, none of the time	8 (11)	0	8 (5)
NA#	6 (8)	0	6 (3)
Do you trust human health professionals’ decisions about which medical treatments are best for you?			
Yes, all of the time	48 (63)	61 (62)	109 (62)
Most of the time	16 (21)	28 (28)	44 (25)
Sometimes	2 (3)	7 (7)	9 (5)
No, none of the time	5 (7)	1 (1)	6 (3)
NA#	5 (7)	2 (2)	7 (4)
Are you satisfied with the care you receive from medical professionals in general?			
Yes, all of the time	48 (63)	61 (62)	109 (62)
Most of the time	13 (17)	28 (28)	41 (23)
Sometimes	4 (5)	9 (9)	13 (7)
No, none of the time	6 (8)	1 (1)	7 (4)
NA#	5 (7)	0	5 (3)
Are you satisfied with the care your pet receives from veterinary professionals at this clinic?			
Yes, all of the time	69 (91)	82 (83)	151 (86)
Most of the time	4 (5)	10 (10)	14 (8)
Sometimes	0	3 (3)	3 (2)
No, none of the time	1 (1)	0	1 (1)
NA#	2 (3)	4 (4)	6 (3)
Do you trust veterinarians’ decisions about which medical treatments are best for your pet?			
Yes, all of the time	67 (88)	76 (77)	143 (82)
Most of the time	5 (7)	21 (21)	26 (15)
Sometimes	0	1 (1)	1 (1)
No, none of the time	1 (1)	0	1 (1)
NA#	3 (4)	1 (1)	4 (2)
Are you satisfied with the care your pet receives from veterinarians in general?			
Yes, all of the time	66 (86)	67 (68)	133 (76)
Most of the time	7 (9)	23 (23)	30 (17)
Sometimes	0	6 (6)	6 (3)
No, none of the time	1 (1)	0	1 (1)
NA#	3 (4)	3 (3)	6 (3)

NA#, left blank. Statistical significance was determined using a chi-square test to compare responses between Clinic A and Clinic B.

* *P* < .001.

** *P* < .0129.

*** *P* < .00007.

For Table 2, a chi-square test was conducted to analyze the distribution of yes/no responses to 3 key questions: (1) “Did you receive any health screenings during your visit today?” (2) “Did you receive any new health information, such as being informed of high blood pressure?” and (3) “Were you provided with follow-up information or resources to support your health?” The test yielded statistically significant *P*-values of <.0001, .0129, and .000007, respectively, indicating a meaningful impact on patient engagement and health education. Additionally, independent t-tests were performed on the remaining Likert-scale survey questions in Tables 2 and 3 to assess differences in patient experience and satisfaction; however, no statistically significant differences were observed. As this study is exploratory and hypothesis-generating in nature, adjustments for multiple comparisons were not applied.

**Table 3.** Responses to Statements and Questions Regarding Human-animal Bond From 175 Individuals Seen at 2 One Health Clinics Between August 2023 to November 2024.

	Clinic A (N = 76) (%)	Clinic B (N = 99) (%)	Total (N = 175) (%)
I consider my pet to be a part of my family.			
Strongly agree	61 (80)	92 (93)	153 (87)
Agree	13 (17)	6 (6)	19 (11)
Neither agree/disagree	0	0	0
Disagree	1 (1)	0	1 (1)
Strongly disagree	0	0	0
NA#	1 (1)	1 (1)	2 (1)
My pet(s) helps me to be more active (i.e., exercise, go for walks, etc.).			
Strongly agree	42 (55)	66 (67)	108 (62)
Agree	24 (32)	19 (19)	43 (25)
Neither agree/disagree	5 (7)	8 (8)	13 (7)
Disagree	3 (4)	3 (3)	6 (3)
Strongly disagree	1 (1)	3 (3)	4 (2)
NA#	1 (1)	0	1 (1)
My pet(s) helps me feel less depressed/ anxious.			
Strongly agree	51 (67)	69 (70)	120 (69)
Agree	18 (24)	24 (24)	42 (24)
Neither agree/disagree	3 (4)	4 (4)	7 (4)
Disagree	3 (4)	1 (1)	4 (2)
Strongly disagree	0	0	0
NA#	1 (1)	1 (1)	2 (1)
My pet(s) makes me feel less lonely.			
Strongly agree	47 (62)	75 (76)	122 (69)
Agree	21 (28)	19 (19)	40 (23)
Neither agree/disagree	5 (7)	4 (4)	9 (5)
Disagree	1 (1)	1 (1)	2 (1)
Strongly disagree	1 (1)	0	1 (1)
NA#	1 (1)	0	1 (1)
Concerns about providing care for my pet(s) cause me stress.			
Strongly agree	11 (14)	30 (30)	41 (23)
Agree	16 (21)	28 (28)	44 (25)
Neither agree/disagree	14 (18)	18 (18)	32 (18)
Disagree	13 (17)	18 (18)	31 (18)
Strongly disagree	21 (28)	4 (4)	25 (14)
NA#	1 (1)	1 (1)	2 (1)

NA#, left blank.

Participants were asked about their bond with their pets (Table 3). Most considered their pet a family member (Clinic A: 80%, Clinic B: 93%) and felt their pet encouraged physical activity (Clinic A: 87%, Clinic B: 85%). A majority also reported that their pet helped reduce feelings of depression, anxiety, and loneliness. However, responses were mixed across both clinics regarding whether caring for their pet caused stress.

**Figure 2.**
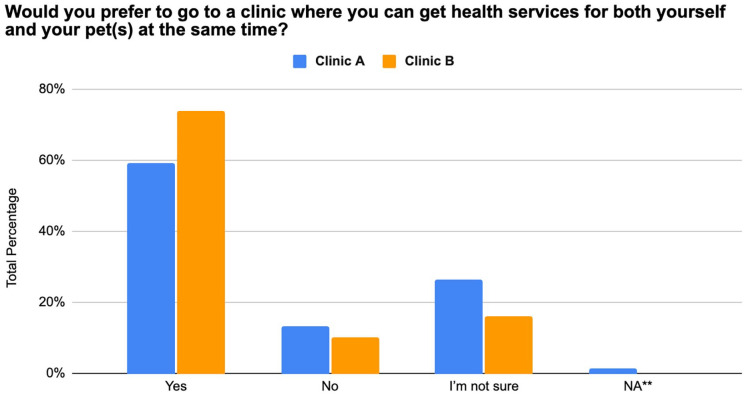
Interest in attending clinics with integrated human and pet health services from 175 individuals between August 2023 and November 2024.

**Figure 3.**
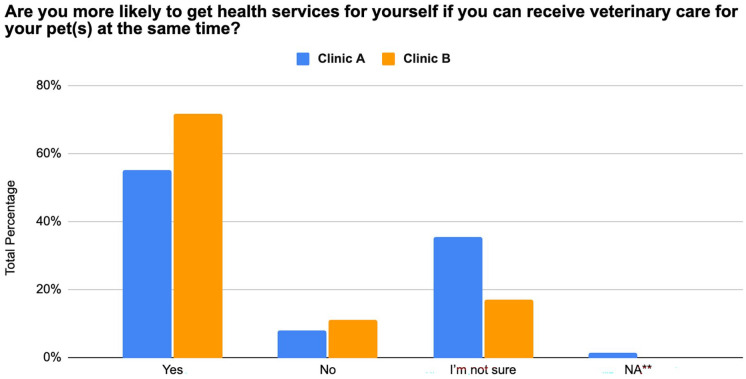
Interest in attending clinics with integrated human and pet health services from 175 individuals between August 2023 and November 2024.

The majority of participants from both OHCs responded that they were more likely to get healthcare services for themselves if they could receive care for their pet simultaneously and that they would prefer to attend clinics that have this set-up (Clinic A = 56% and 60%, Clinic B = 72% and 74%) (Figures 2 and 3).

## Discussion

A key difference between the 2 OHCs was the utilization of human healthcare services. At Clinic B, 91% of participants reported receiving a health screening compared to only 32% at Clinic A—a statistically significant difference (*P* < .00001). Additionally, a greater proportion of individuals at Clinic B (48%) reported learning new information about their health, compared to 31% at Clinic A (*P* < .00007). These findings highlight a substantially higher level of engagement with preventive health services at Clinic B. This result likely reflects the clinic structure, since Clinic B requires engagement with healthcare professionals as a prerequisite for receiving free veterinary services, while Clinic A makes this engagement optional for clinic attendees. Mandatory engagement with healthcare services at OHCs may encourage individuals to seek healthcare. Medical professional availability is another important factor since Clinic B reports having medical professionals at all clinics, a limitation for Clinic A due to the inconsistent availability of medical professionals at all clinics. Consistent medical professional presence at OHCs may increase engagement with human healthcare services, which supports individuals in addressing their health needs while fostering trust in medical professionals. Requiring some level of engagement with medical professionals at OHCs may encourage engagement with human healthcare services. Additionally, a notable percentage of individuals reported that they intended to follow up with their healthcare provider after their visit (Clinic A = 58%, Clinic B = 75%). This suggests that OHCs not only provide immediate access to healthcare but may also help facilitate continued medical care engagement beyond the clinic. However, additional research is needed to determine whether this model translates to sustained healthcare engagement.

Trust in medical professionals plays a crucial role in engagement. Participants at both OHCs reported high levels of trust and satisfaction toward medical and veterinary healthcare professionals at these clinics and in general (Table 2). These findings align with prior research suggesting that OHCs may foster trust between healthcare professionals and historically underserved communities, contributing to improved health outcomes.^24^ The majority of clinic attendees reported being from racial/ethnic, geographic, and socioeconomic groups that are historically underserved, particularly in terms of access to both medical and veterinary care.^2,9,25-27^ Distrust in healthcare and its impact on health outcomes are well-documented within these underserved populations,^28-32^ making the high levels of trust and satisfaction among attendees at OHCs particularly meaningful. This suggests that such models may help foster connection, build credibility, and support ongoing engagement.

In addition to socioeconomic and racial disparities, another factor that may influence human healthcare engagement is the HAB.^33-35^ Overall, participants’ responses indicated a strong bond between their pet and themselves. The majority of attendees at both clinics considered their pet a family member (Clinic A = 81%, Clinic B = 93%) and agreed that their pet had a positive impact on their physical and mental well-being (Table 3). However, concerns regarding pet care-related stress varied, with Clinic B participants reporting higher stress (Clinic A = 35%, Clinic B = 58%). This difference may be influenced by a variety of factors, such as economic resources, individuals’ mental state, pet type and quantity, an individual’s relationship with their pet (e.g., companionship vs working), and health issues of both individuals and their pet.^36-38^ Overall, these findings emphasize the perceived emotional, psychological, and physical benefits of pet ownership.

The primary reason for attending an OHC was to receive veterinary services (Clinic A = 91%, Clinic B = 75%). This highlights the key role that pet care plays in motivating individuals to engage with OHCs. However, it is also worth noting that some participants (Clinic A = 9%, Clinic B = 22%) reported seeking access to both veterinary and human healthcare services, indicating that some clinic attendees value and utilize the integrated OHC care model. Further supporting this idea, the majority of participants responded that they were more likely to go to clinics that allowed them to get simultaneous care for themselves and their pets. This preference for an integrated OHC model (Clinic A = 56%-60%, Clinic B = 72%-74%) supports the idea that veterinary care may serve as an entry point for human healthcare engagement.

## Limitations

This study had several limitations. First, convenience sampling and collecting data from only 2 clinics limit the generalizability of these findings beyond their specific populations. Second, self-reported responses may reflect recall or social desirability bias, potentially inflating satisfaction or follow-up intent. Third, differences in clinic procedures and provider availability affected consistency in participant experiences. Fourth, the cross-sectional design captures a single time point, preventing assessment of long-term healthcare engagement. Lastly, the survey did not assess environmental health factors or their impact on services provided to humans and animals. Given that both clinics serve disadvantaged populations frequently exposed to environmental hazards such as pesticides, smog, and extreme heat, incorporating questions on environmental exposures and occupation could strengthen the One Health model these clinics aim to uphold.

## Conclusion

Our findings suggest that OHCs can improve access to human healthcare in underserved communities by using veterinary services as an entry point, even when variable models are used. The strong human-animal bond (HAB) reported by participants highlights how pets motivate individuals to seek care. High levels of trust and satisfaction indicate that OHCs build confidence in both medical and veterinary services, encouraging continued use. Differences between clinics show that engagement in human healthcare varies with clinic structure; consistent medical staffing and requiring minimal human health engagement for veterinary care may increase participation and support better health outcomes.

Future efforts should determine the variable OHC models that could be used and expand the most effective OHC models to diverse regions and populations. Long-term studies are needed to assess sustained healthcare engagement and outcomes for both the people and pets. Further research on the role of the HAB in motivating care-seeking could deepen understanding of OHCs as a public health tool. Comparing health outcomes and preventive care uptake between OHC attendees and those using traditional clinics will be key to evaluating their impact.

The OHC model represents a powerful, community-centered approach to advancing health equity—bridging gaps in care, strengthening trust, and meeting both human and animal health needs in a single, accessible setting.
